# Supplementary material for: The interactome of intact mitochondria by cross-linking mass spectrometry provides evidence for coexisting respiratory supercomplexes
Source: Mol Cell Proteomics. 2017 Dec 8;17(2):216–32. doi: 10.1074/mcp.RA117.000470 (PMC5795388; doi:10.1074/mcp.RA117.000470)
Supplement: Supplemental Data [file supp_RA117.000470_133922_0_supp_23978_fzffwf.zip › spectra_annotation/mito_spectra_annotation/369-1-5-1-3-1.pdf]

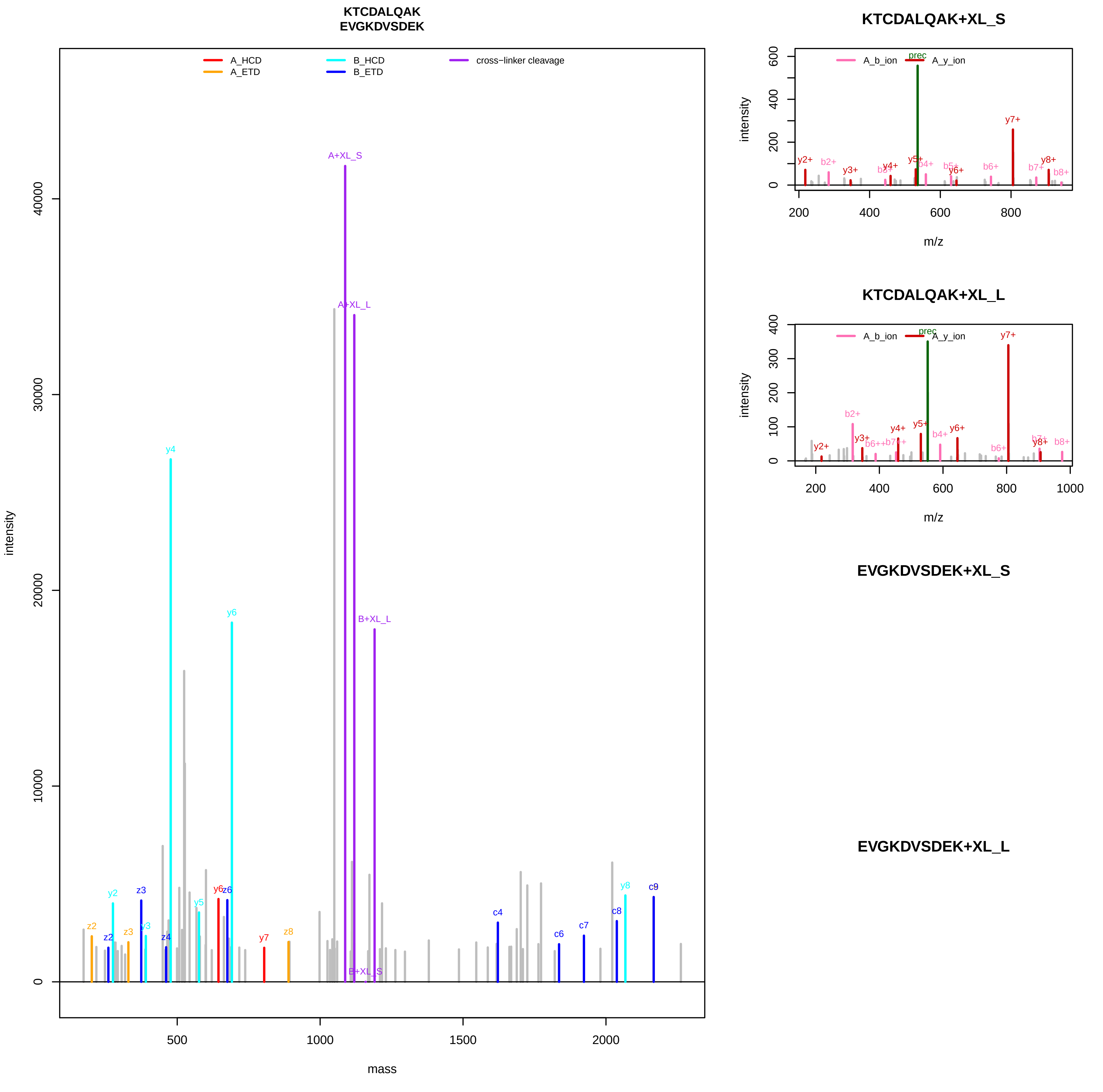

KTCDALQAK
EVGKDVSDEK
A_HCD
A_ETD
B_HCD
B_ETD
cross−linker cleavage
A+XL_S
A+XL_L
B+XL_L
B+XL_S
y4
y6
y2
z2
z3
y3
z4
y5
y6
z6
y7
z8
c4
c6
c7
c8
y8
c9
intensity
mass
0
10000
20000
30000
40000
500
1000
1500
2000
KTCDALQAK+XL_S
A_b_ion
A_y_ion
prec
y2+
b2+
y3+
b3+
y4+
y5+
b4+
b5+
y6+
b6+
y7+
b7+
y8+
b8+
intensity
m/z
0
200
400
600
800
KTCDALQAK+XL_L
A_b_ion
A_y_ion
prec
y7+
b2+
y2+
y3+
b6++
b7++
y4+
y5+
b4+
y6+
b6+
b7+
y8+
b8+
intensity
m/z
0
100
200
300
400
600
800
1000
EVGKDVSDEK+XL_S
EVGKDVSDEK+XL_L
